# Delivery of miR-25802 via Small Vesicles Protects Against Mitochondrial Injury, Oxidative Stress, and Neuroinflammation in Alzheimer’s Disease

**DOI:** 10.1007/s12035-026-05889-7

**Published:** 2026-04-23

**Authors:** Hamit Çelik, Elif Dalkılınç, Şeyma Aydın, Oğuz Çelik, Sefa Küçükler, Ahmet Topal, Ramazan Akay, Sinan Gönüllü, Mustafa Onur Yıldız, Bülent Alım, Selçuk Özdemir

**Affiliations:** 1 Department of Neurology, Private Buhara Hospital, Erzurum, Türkiye; 2 Department of Biochemistry, Faculty of Veterinary Medicine, Atatürk University, Erzurum, Türkiye; 3 Department of Genetics, Faculty of Veterinary Medicine, Atatürk University, Erzurum, Türkiye; 4 Savur Prof. Dr. Aziz Sancar District State Hospital, Mardin, Türkiye; 5 Department of Basic Sciences, Faculty of Fisheries, Atatürk University, Erzurum, Türkiye; 6 Department of Neurology, Eskisehir City Hospital, Eskişehir, Türkiye; 7 Department of Neurology, Bursa City Hospital, Bursa, Türkiye; 8 Department of Neurology, Faculty of Medicine, Samsun University, Samsun, Türkiye

**Keywords:** Alzheimer’s Disease, MicroRNA-25802, Mitochondrial Dysfunction, Neuroinflammation, Oxidative Stress

## Abstract

Mitochondrial dysfunction, oxidative stress, and neuroinflammation play a critical role in the occurrence and progression of Alzheimer's disease (AD). MicroRNAs (miRNAs) have been studied recently as potential therapeutic approaches for AD. In this study, we examined the function and underlying mechanism of microRNA-25802 (miR-25802), a newly discovered miRNA in an AD model. In order to evaluate the levels of oxidative stress, mitochondrial damage and neuroinflammation in neuroblastoma cells, four experimental groups were created: control group (neuroblastoma cells, SH-SY5Y), amyloid beta (Aβ)-induced neuroblastoma cells (SY5Y-Aβ), small extracellular vesicles (sEVs)-only group and miR-25802–loaded small extracellular vesicles (sEV-miR25802) administered group. Neuroinflammation, oxidative stress, mitochondrial damage, tau hyperphosphorylation, and Aβ accumulation were evaluated in Aβ-induced neuroblastoma cells. Oxidative stress was analyzed by measuring reactive oxygen species (ROS), malondialdehyde (MDA), lactate dehydrogenase (LDH), superoxide dismutase (SOD), and glutathione peroxidase 1 (GPX1). Inflammatory markers such as tumor necrosis factor-alpha (TNF-α), intercellular adhesion molecule 1 (ICAM1), and brain-derived neurotrophic factor (BDNF) mRNA levels, a neurotrophic factor, were evaluated by RT-qPCR. Neurofilament light chain (NfL), vascular endothelial growth factor-A (VEGF-A), macrophage migration inhibitory factor (MIF), monocyte chemoattractant protein-1 (MCP-1) and cytochrome c (Cyt-c), mitochondrial transcription factor A (TFAM), PTEN-induced kinase 1 (PINK1) and dynamin-1-like protein (DNM1L) protein levels were determined by ELISA. Mechanistically, sEV-miR25802 were shown to provide anti-inflammatory and neuroprotective effects by regulating neuroinflammation, mitochondrial dysfunction, and oxidative stress. These findings reveal the regulatory role of miR-25802 on neuroinflammation, mitochondrial damage, and oxidative stress and suggest that it may be a potential therapeutic target for AD.

**Graphical Abstract:**

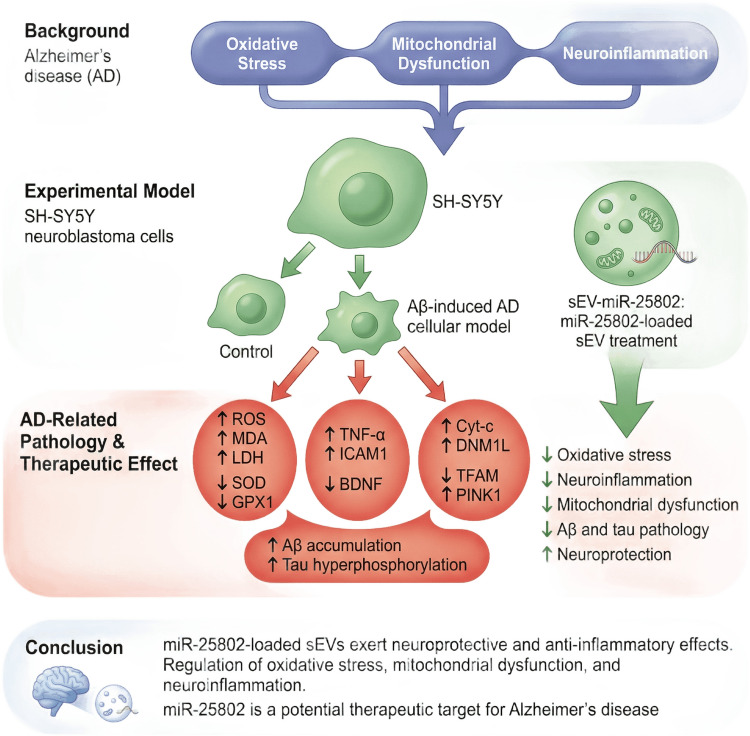

**Supplementary Information:**

The online version contains supplementary material available at 10.1007/s12035-026-05889-7.

## Introduction

Alzheimer's disease (AD) is a neurodegenerative disorder that accounts for approximately 70% of all dementia cases [1] and progressively affects memory, behavior, and other cognitive functions over time [2, 3]. The development of tau protein tangles and amyloid beta (Aβ) plaques, as well as cognitive decline and dementia, have all been connected to the disease's etiology [4]. The accumulation of Aβ peptides and neurofibrillary tangles in the brain triggers oxidative stress and neuroinflammation, leading to synaptic dysfunction, memory impairment, and ultimately neurodegeneration [5, 6]. Two cytotoxic proteins in the brain, Aβ peptides and phosphorylated tau (p-Tau), activate the immune system by stimulating astrocytes and microglia. The immune activation induced by these proteins leads to the release of pro-inflammatory cytokines and the development of neuroinflammation [7]. In addition, oxidative stress caused by free radicals produced abnormally during cellular metabolism promotes the development of AD [8]. Oxidative stress, which damages cells and causes disease progression, results from an imbalance between prooxidants and antioxidant defense mechanisms [9, 10]. Despite extensive research aimed at identifying therapeutic targets, the molecular and cellular mechanisms underlying AD pathogenesis remain unclear, making it difficult to develop effective treatment strategies.

MicroRNAs (miRNAs) are tiny, non-coding RNA molecules with an average length of 22 nucleotides [11]. Many miRNAs undergo transcription to form primary miRNAs (pri-miRNAs). The Drosha enzyme subsequently breaks down the resultant pri-miRNAs into precursor miRNA (pre-miRNA) fragments that are roughly 70 nucleotides long [12]. The pre-RNA is subsequently transferred to the cytoplasm where it is processed by the RNase III endonuclease Dicer to create a double-stranded RNA molecule [13]. The complementary strand of this duplex is broken while the functional strand is loaded onto the Argonaute protein within the RNA-induced silencing complex (RISC) [14]. After being integrated into the RISC, mature miRNAs interact with target mRNAs to form active RISC-miRNA complexes that alter gene expression [15]. MiRNAs regulate gene expression in two main ways: either they bind entirely to the 3' untranslated region (3' UTR) of target mRNAs, which causes mRNA degradation and inhibits gene expression, or they bind partially to the 3' UTR to suppress translation, which lowers gene expression [16]. Through this mechanism, miRNAs play regulatory roles in cellular metabolism, proliferation, differentiation, developmental processes, apoptotic cell death, viral infections, and the molecular pathogenesis of various diseases [17].

Extracellular vesicles (EVs) are membrane-enclosed, nano-sized vesicles that play a crucial role in intracellular communication under both healthy and pathological conditions [18, 19]. EVs are primarily categorised into three groups based on their biophysical characteristics, size, cellular origin, and biogenesis mechanisms: apoptotic bodies, microvesicles, and exosomes [20]. Microvesicles are large vesicles (100–1000 nm in diameter) produced by budding from the cell membrane, whereas exosomes are formed from the endosomal system and then secreted by cells into the extracellular fluid as vesicles with a diameter of 30–100 nm. Apoptotic bodies, on the other hand, are produced during the process of programmed cell death and range in diameter from 1000 to 5000 nm [21].

EVs deliver bioactive molecules, including proteins, peptides, and various types of RNA, to target cells [22]. In particular, small extracellular vesicles (sEVs) can exert broad regulatory effects on cellular activity through their cargo, which includes small non-coding RNAs such as miRNAs and piRNAs. These small RNA molecules, particularly through RNA silencing mechanisms, modulate the expression of target genes, thereby influencing the phenotypic and functional characteristics of recipient cells [23].

Interest in the roles of exosomes has increased as mRNA and miRNAs in exosomes are transferred into cells [22–24]. The intricate pathophysiology of AD is mostly influenced by oxidative stress, mitochondrial failure, and neuroinflammation [25, 26]. Therefore, the delivery of therapeutic miRNAs via EVs is considered a promising strategy for developing specific and biologically meaningful interventions in target cells.

This study aimed to investigate the potential protective effects of miRNA-25802 (miR-25802) delivered via sEVs on mitochondrial damage, oxidative stress, and neuroinflammation in AD.

## Material and Methods

### Characterization of Milk-Derived sEV

Sterile cow's milk was obtained from Atatürk University Farm and stored at + 4 °C before use. Milk samples confirmed to be sterile by microbiological tests were used in the study. For the isolation of the sEV fraction, sequential centrifugation and ultracentrifugation steps were performed according to the protocol described by Del Pozo-Acebo et al. [27]. This method enabled the isolation of sEVs of high purity from the milk components.

In short, 1 mL of milk sample was centrifuged at 13,000 × g for 30 min at 4 °C using a JLA-16.250 rotor in an Avanti J-26XPI centrifuge (Beckman Coulter, Brea, CA, USA). The resulting supernatant was subjected to a second centrifugation at 35,000 × g for 60 min at 4 °C to remove casein and other large protein aggregates. The clear supernatant (skim milk) obtained after this process was ultracentrifuged at 100,000 × g for 105 min at 4 °C using an Optima L-90 K ultracentrifuge (Beckman Coulter) with a 50.2 Ti rotor, and EVs were pelletized. After discarding the supernatant, the pellet containing EVs was resuspended in 25 mL phosphate-buffered saline (PBS).

A second ultracentrifugation was carried out at 100,000 × g for 105 min at 4 °C in order to wash and concentrate the EV pellets. After resuspending the sEV-enriched pellet in 700 µL of PBS to create a homogenous suspension, the bigger particles were filtered out using a 0.22 µm Millex®-GP syringe filter (Merck Millipore, Burlington, MA, USA). For additional purification, the filtered sEVs (700 µL) were submitted to size exclusion chromatography (SEC) using an Izon qEV column (Izon Science, USA); this step was carried out in accordance with the protocol given by [28]. First, 10 mL of cold PBS was used to equilibrate the SEC column. The filtered sEV suspension was added after column activation, and elution was carried out using an extra 10 mL of PBS. The first 3 mL of eluate were thrown away. The 2 mL fractions that followed were collected into Eppendorf tubes. Fractions containing sEV were centrifuged at 4,000 × g for 40 min at 20 °C using the Vivaspin concentrator (Sartorius, USA), yielding a final volume of approximately 2 mL [29]. The MISEV 2018 standards were followed in the characterization and classification of EVs [30].

### Nanoparticle Tracking Analysis (NTA)

The particle count and size distribution of the sEVs were ascertained using NTA. The NanoSight NS300 system (Malvern Technologies, Malvern, UK) was the apparatus utilized. It has a high-sensitivity scientific CMOS camera and a 488 nm laser. Samples were diluted 1:500 in serum-free PBS (Gibco, Waltham, MA, USA) per the manufacturer's instructions. During analysis, samples were evaluated under continuous flow conditions (flow rate = 50) at a temperature range of 43–45 °C; 15 video recordings, each lasting 60 s, were obtained using 14 different camera angles. NTA 3.1.54 software was used to analyze the acquired images; the bin size was set to 2 and the detection threshold to 5 [31].

### Dynamic Light Scattering (DLS)

The Zetasizer Nano ZS device (Malvern Instruments, Malvern, UK) was used to measure the hydrodynamic diameter and size distribution of sEV samples using DLS. The vesicle suspensions were diluted 1:250 in 1 mL of 1 × PBS before being measured, and any large aggregates were removed using a 0.45 µm pore syringe filter. All analyses were performed at 25 °C in accordance with the manufacturer's instructions [32].

### Imaging using Transmission Electron Microscopy (TEM)

At the initial stage, isolated sEV samples were applied to formvar/carbon-coated copper EM grids. For each grid, 10 μL of sEV suspension was used, and this incubation process lasted 20 min. The grids were then rinsed three times with PBS to remove loose particles and fixed for 10 min in a 2.5% glutaraldehyde solution. After fixation, the grids were washed with ultrapure water and stained for contrast with 2% uranyl acetate solution. After the staining step, the grids were dried at room temperature for 20 min. A Tecnai 120 FEI microscope was used to perform transmission electron microscopy at an accelerating voltage of 120 kV [31].

### Active Loading of miR-25802 into sEVs

The synthetic miR-25802 mimic used in this study was supplied by Thermo Fisher Scientific (Waltham, MA, USA). By incubating 200 µg of sEV protein in 1 mL of 1 × PBS with 1000 µg of miR-25802 mimic and 0.2% (w/v) saponin, miR-25802 was actively loaded int sEVs. The saponin used in the loading procedure was provided from Merck (Darmstadt, Germany).

The identical procedure was used to create control samples, but no miR-25802 mimic was added. Samples were gently vortexed and incubated for approximately 10 s. Following incubation, each sample was purified using PD-10 size-exclusion columns (Cytiva, USA). After purification, the first 1.5 mL of the eluate containing the EV fractions were collected as previously described [28].

### Validation of Exogenous miR-25802 Loading in sEVs by Real-Time Quantitative Polymerase Chain Reaction (RT-qPCR)

Quantitative RT-qPCR analysis of RNAs extracted from isolated vesicle fractions verified the successful encapsulation of miR-25802 exogenously supplied to sEVs. Only RNase and RNase + membrane disrupting treatments were used during pre-loading. The study also used input samples, along with miRNA-loaded, mock-loaded, and free miRNA-only controls. Additionally, the same amount of a non-mammalian spike-in miRNA was added to each sample to assess the consistency of RNA extraction. A miRNA-specific reverse transcription technique (ideally stem-loop or polyA/universal RT) was used to create cDNA in order to detect miR-25802. Subsequently, RT-qPCR analysis was used to quantitatively determine miR-25802 levels using commercially available or probe-validated primer/probe sets. To determine absolute copy number, quantification was facilitated using synthetic miR-25802 standard curves. Data were presented as both copy number per particle and relative fold change. The increase in miR-25802 levels observed in loaded sEV samples indicates loading efficiency and encapsulation of the miRNA. However, combined application of RNase and membrane disruption resulted in decreased miR-25802 levels, confirming that miRNA was largely loaded into vesicles. The obtained Ct and copy number data were subjected to statistical analysis.

### Cell Culture

The American Type Culture Collection (ATCC, Manassas, VA, USA) provided the SH-SY5Y human neuroblastoma cell line. SH-SY5Y human neuroblastoma cells were chosen as a well-established in vitro neuronal model due to their sensitivity to Aβ–induced oxidative stress and mitochondrial dysfunction, allowing the investigation of early AD–related cellular alterations under controlled conditions. Dulbecco's Modified Eagle Medium (DMEM; Gibco, MA, USA) with 10% fetal bovine serum (FBS; Gibco, MA, USA) and 1% penicillin–streptomycin was used to cultivate the cells. Cell cultures were maintained at 37 °C with 5% CO_2_ in a humidified incubator. SH-SY5Y cells were treated with Aβ (C008-4, Meilunbio, Dalian, China) for 48 h to produce SH-SY5Y-Aβ, an in vitro model of AD [33].

### Cellular Viability

The 3-(4,5-dimethylthiazol-2-yl)−2,5-diphenyltetrazolium bromide (MTT) assay (Merck, Germany) was used to evaluate the in vitro cytotoxicity of miR-25802–loaded small extracellular vesicles (sEV-miR25802) in SH-SY5Y neuroblastoma cells at 24 and 48 h. Cells were seeded into 96-well plates at a density of 1 × 10^4^ cells per well and incubated at 37 °C in a humidified atmosphere containing 5% CO₂.

Cells were treated with control sEVs or sEV-miR25802 at concentrations of 0, 0.5, 1, 2.5, 5, and 10 µg/mL. At each experimental time point (24 and 48 h), 10 µL of MTT reagent (Cat. No. M5655, Sigma-Aldrich) was added to each well and incubated for 2 h at 37 °C. The culture medium was then carefully removed, and the resulting formazan crystals were solubilized with 100 µL of dimethyl sulfoxide.

Absorbance was measured at 540 nm using a BIO-RAD 680 XR microplate reader. All experimental conditions were performed in quadruplicate. Dose optimization analysis demonstrated that 10 µg/mL of sEV-miR25802 induced cytotoxic effects, whereas 5 µg/mL was identified as the optimal non-toxic concentration and was therefore used for subsequent experiments [34].

### Measurement of Intracellular Reactive Oxygen Species (ROS)

A single-step fluorometric test kit was used to quantify the intracellular ROS (Cat. No: ab11385, Abcam) levels in each experimental group. 100 μL of SH-SY5Y cells were planted into transparent-bottomed, black 96-well plates. The plates were incubated for 24 h at 37 °C with 5% CO_2_ after the cells were seeded. The primary reaction mixture was added to each well at the conclusion of the incubation period, and the plates were then re-incubated under the same circumstances. After that, the fluorescent signal was measured at 520 nm for excitation and 605 nm for emission. The ROS level of the cells in the control group was accepted as a 100% reference value; the ROS levels of the experimental groups were calculated as a percentage relative to this reference.

### Enzyme-Linked Immunosorbent Assay (ELISA)

In human samples, the levels of total Tau (MBS812766), phosphorylated Tau-181 (pTau-181) (MBS744973), phosphorylated Tau-217 (pTau-217) (MBS1608795), Aβ 1–40 (MBS2506221), and neurofilament light chain (NfL) (MBS9399603) as well as the oxidative stress markers malondialdehyde (MDA) (MBS7606463), superoxide dismutase (SOD) (MBS009535), lactate dehydrogenase (LDH) (MBS009535), and glutathione peroxidase 1 (GPX1) (MBS026180), were quantitatively measured using commercial human ELISA kits (MyBioSource, USA) according to the manufacturers’ protocols. Additionally, biochemical markers related to neuroinflammation and mitochondrial damage, including macrophage migration inhibitory factor (MIF) (E-EL-H6170, Elabscience), vascular endothelial growth factor-A (VEGF-A) (E-EL-H0111, Elabscience), monocyte chemoattractant protein-1 (MCP-1) (KE00277, Proteintech), cytochrome c (Cyt-c) (MBS162423, MyBioSource), mitochondrial transcription factor A (TFAM) (MBS2020891, MyBioSource), PTEN-induced kinase 1 (PINK1) (MBS2124222, MyBioSource), dynamin-1-like protein (DNM1L) (MBS2124218, MyBioSource), complexin 2 (CPLX2) (MBS450651, MyBioSource), and receptor tyrosine kinase-like orphan receptor 1 (ROR1) (MBS2000330, MyBioSource) were also analyzed using commercial human ELISA kits according to the manufacturers’ instructions. The resulting colorimetric changes were measured at 450 nm using a microplate reader.

### Real-Time Quantitative Polymerase Chain Reaction (RT-qPCR)

Using QIAzol Lysis Reagent (Cat. No: 79306, Qiagen, Germany), total RNA, including small RNAs, was extracted from SH-SY5Y cells in accordance with the manufacturer's instructions. Complementary DNA (cDNA) analysis was performed using the Omniscript RT Kit (Cat. No: 205113, Qiagen, Germany) according to the manufacturer's instructions. The same kit and a stem-loop RT primer specific to miR-25802 were used for reverse transcription in order to analyze miRNA expression.

The QuantiNova SYBR Green PCR Kit (Cat. No: 208052, Qiagen, Germany) was used for quantitative real-time PCR studies on the Rotor-Gene Q MDx 5plex HRM (Qiagen, Germany) apparatus. Initial denaturation at 95 °C for 5 min, 40 cycles of denaturation at 95 °C for 10 s, and annealing/extension at 60 °C for 30 s comprised the thermal cycling protocol. Melting curve analysis was done following each run to verify the amplification's specificity.

The primer sequences for the target genes brain-derived neurotrophic factor (BDNF), intercellular adhesion molecule 1 (ICAM1), tumor necrosis factor alpha (TNFα), miR-25802, and the internal controls glyceraldehyde-3-phosphate dehydrogenase (GAPDH) (for mRNA) and U6 snRNA (for miRNA) are presented in Table 1. The primer sequences used in BDNF [35] and TNF-α [36] gene expression analyses were selected based on previously published studies, and their specificity to the Homo sapiens genome and lack of non-specific amplification were confirmed using NCBI Primer-BLAST. Primer specificity was confirmed by single peak melting curves and agarose gel electrophoresis of the amplified products. In each experiment, template control (NTC) or reverse transcriptase negative control (− RT) samples were used to control for potential contamination or genomic DNA amplification.

**Table 1:** Primer sequences and informations

**Gene Name**	**Primer Sequences**	**Product Size (bp)**	**Accession Number**
BDNF	**F:** TGCAGGGGCATAGACAAAAGG **R:** CTTATGAATCGCCAGCCAATTCTC	110	NM_001143812.2
ICAM1	**F:** AGAGACCCCGTTGCCTAAAA **R:** CAGTACACGGTGAGGAAGGT	153	NM_000201.3
TNF-α	**F:** GCTGCACTTTGGAGTGATCG **R:** GAGGGTTTGCTACAACATGGG	140	NM_000594.4
GAPDH	**F:** AGGTCGGAGTCAACGGATTT **R:** ATCGCCCCACTTGATTTTGG	200	NM_001357943.2
miR-25802	TCACGGATACAGCCTCCTTTGGGA	-	-
U6 snRNA	CGCAAGGATGACACGCAAATTC	-	-

The 2^ − ΔΔCt technique was used to calculate relative gene expression levels [37]. The GAPDH reference gene was used to normalize mRNA expression levels, while U6 snRNA was used to normalize miR-25802 expression levels. Melting Curve graphics are provided at Supplementary Fig. 1.

### Western Blot Analysis

Following treatment, the cells were lysed using RIPA buffer containing protease and phosphatase inhibitors. Protein concentrations were measured using the BCA assay. Equal amounts of protein (50 μg) were separated on 10% SDS-PAGE gels and transferred to PVDF membranes. The membranes were blocked in TBST containing 5% non-fat milk at room temperature for 1 h, then incubated overnight at 4 °C with primary antibodies against BDNF (Cat. No.: ab108319, 1:1000, Abcam, UK) and β-actin (Cat. No.: bs-0061R, 1:1000, Bioss, USA). After washing, the membranes were incubated at room temperature for 1 h with HRP-conjugated secondary antibodies (Cat. No.: sc-2357, 1:5000, Santa Cruz Biotechnology, USA). Protein bands were visualized using the Thermo iBright Imaging System and quantitatively assessed using iBright Analysis Software.

### Statistical Analysis

Statistical analysis was performed using R (version 4.5.1; R Foundation for Statistical Computing, Vienna, Austria). The normality of the data for each group was assessed using the Shapiro–Wilk test. Parametric tests were used for normally distributed data, and non-parametric tests were used for non-normally distributed data. Comparisons between normally distributed groups were evaluated using one-way analysis of variance (ANOVA); when a significant difference was found, Tukey's post hoc test was used for pairwise comparisons. For non-normally distributed data, comparisons between two groups were evaluated using the Mann–Whitney U test, and differences between the four experimental groups were evaluated using the Kruskal–Wallis (KW) test. After the Kruskal–Wallis test, pairwise comparisons were made using the Dunn post hoc test. Results are reported as mean ± standard error (SEM), and a p-value less than 0.05 is considered statistically significant.

## Results

### Efficient and Stable Loading of miR-25802 into sEVs

**Fig. 1:**
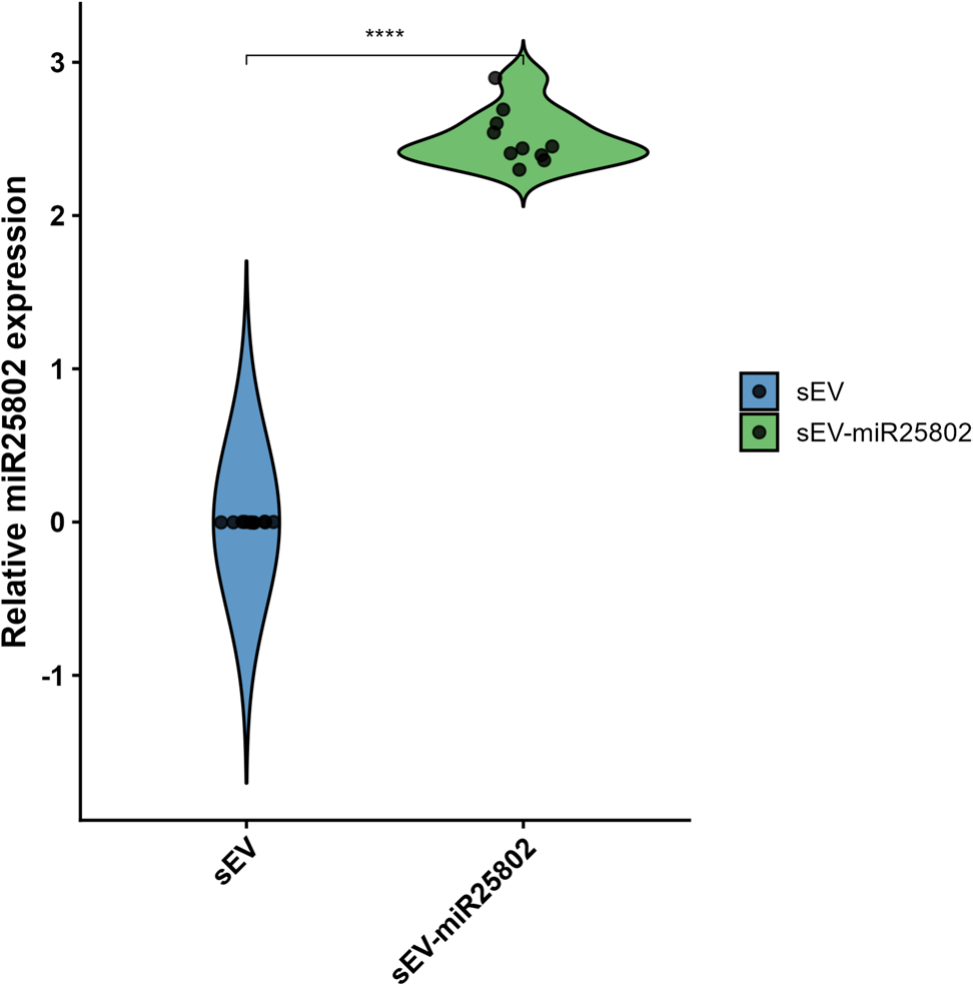
Validation of miR-25802 loading into sEVs: Expression levels in sEV-miR25802 complexes. The mean ± SEM is used to express the data. Statistical significance between two groups was evaluated using the Mann–Whitney U test (*p < 0.05, **p < 0.01, ***p < 0.001, ****p < 0.0001; ns, not significant)

The findings presented in Fig. 1 show that miR-25802 expression was significantly increased in the sEV-miR25802 group compared to sEV (*p* < 0.001). These results confirm that the loading technique used integrates miR-25802 into sEVs, demonstrating that this provides an efficient route for delivering miR-25802 to target cells via sEVs.

### Characterization of sEVs and sEV-miR25802

**Fig. 2:**
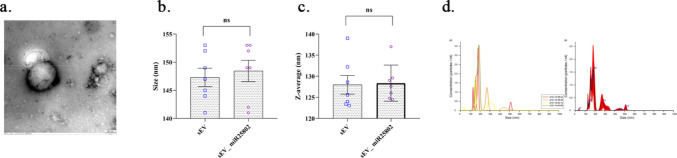
Characterization of sEVs and sEV‑miR25802 complexes: Transmission Electron Microscopy (TEM) morphology (**a**), Particle size measured by Nanoparticle Tracking Analysis (NTA) (**b**), Z-average hydrodynamic diameter determined by Dynamic Light Scattering (DLS) (**c**) and size distribution and concentration graphs obtained with Nanoparticle Tracking Analysis (NTA) (**d**). The mean ± SEM is used to express the data. One Way ANOVA was used to examine statistical significance (* *p* < 0.05, ** *p* < 0.01, *** *p* < 0.001, ns: not significant)

As shown in Fig. 2, TEM analyses revealed that the vesicles in both groups were structurally intact (Fig. 2a). Furthermore, no significant difference was observed between the sEV and sEV-miR25802 groups in terms of particle size (Fig. 2b) and mean hydrodynamic diameter (Fig. 2c) (p > 0.05). These findings indicate that the physical properties of sEVs were not affected after loading with miR-25802. These results demonstrate that the loading of miR-25802 into sEVs was successfully achieved while maintaining the stability and integrity of the vesicles.

### Cytotoxicity Assessment Using MTT Analysis

**Fig. 3:**
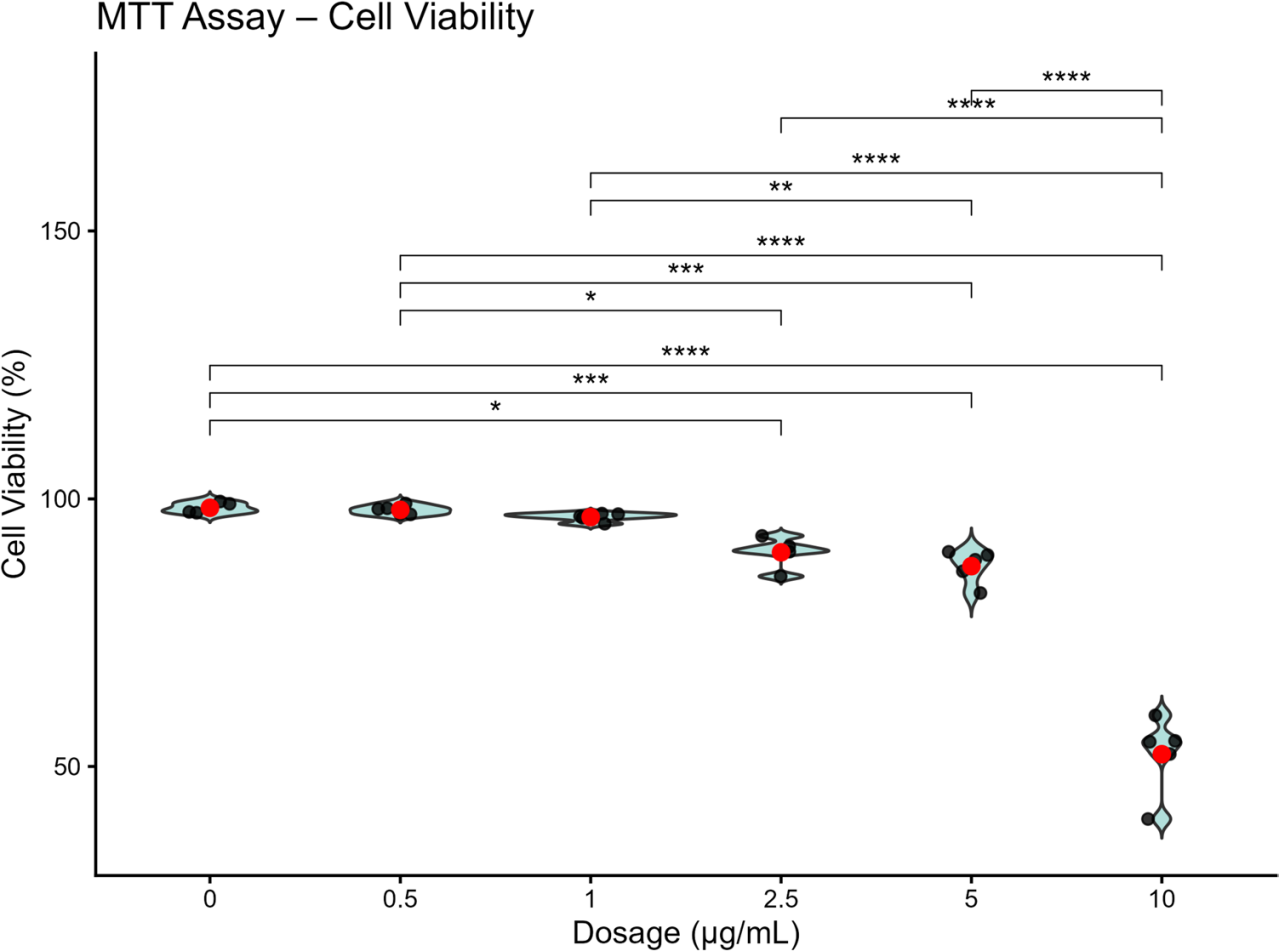
Cytotoxicity assessment of sEV-miR25802 complexes in neuroblastoma cells. Cytotoxic effects of sEV-miR25802 were evaluated by MTT assay across a range of concentrations (0, 0.5, 1, 2.5, 5, and 10 µg/mL). Data are presented as mean ± SEM from 10 biological replicates with 3 technical repeats each. One Way ANOVA was used to examine statistical significance (* *p* < 0.05, ** *p* < 0.01, *** *p* < 0.001, ns: not significant)

The MTT assay was performed to assess the cytotoxicity of sEV-miR25802 complexes in neuroblastoma cells. Cells were exposed to increasing concentrations of sEV-miR25802 (0, 0.5, 1, 2.5, 5, and 10 µg/mL). The results demonstrated that treatment with 10 µg/mL led to a significant reduction in cell viability, indicating cytotoxic effects. In contrast, cell viability at concentrations ranging from 0.5 to 5 µg/mL remained comparable to that of the control group, with no statistically significant changes in metabolic activity (Fig. 3; Supplementary Table 1). Based on these findings, 5 µg/mL was selected as the optimal concentration for sEV-miR25802 administration, as it showed good biocompatibility and did not adversely affect neuroblastoma cell viability.

### Assessment of Oxidative Stress and Cellular Damage

**Fig. 4:**
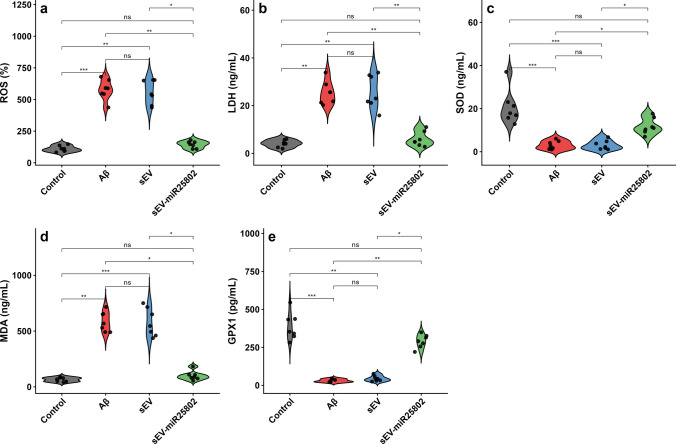
Effects of sEV‑miR25802 on Aβ-Induced Oxidative Stress and Cellular Damage in Neuroblastoma Cells. Reactive oxygen species (ROS) levels (**a**), lactate dehydrogenase (LDH) activity (**b**), superoxide dismutase (SOD) activity (**c**), malondialdehyde (MDA) levels (**d**), and glutathione peroxidase (GPX) activity (**e**). The mean ± SEM is used to express the data. Statistical comparisons were performed using the Kruskal–Wallis test (* *p* < 0.05, ** *p* < 0.01, *** *p* < 0.001, ns: not significant)

Four experimental groups were compared to investigate levels of oxidative stress and cellular damage in neuroblastoma cells: the control group (neuroblastoma cells), Aβ-stimulated neuroblastoma cells, the group treated with sEV alone, and the group treated with sEV-miR25802. Oxidative stress in neuroblastoma cells was assessed by measuring ROS levels (Fig. 4a), LDH activity (Fig. 4b), SOD activity (Fig. 4c), MDA levels (Fig. 4d), and GPX1 activity (Fig. 4e).

In the Aβ-induced group, ROS, MDA, and LDH levels were found to be significantly increased compared to the control group; conversely, SOD and GPX1 activities were significantly decreased (Supplementary Table 2). Furthermore, compared to the Aβ-treated group, the sEV-miR25802-treated group exhibited a significant decrease in ROS, MDA, and LDH levels and a statistically significant increase in SOD and GPX1 activity (*p* < 0.05). These findings suggest that sEV-miR25802 enhances antioxidant defense mechanisms, which is consistent with the neuroprotective effects reported in the literature.

### Evaluation of Neuroinflammation, Glial Activation, and Neuroprotective Responses Using Gene Expression Analysis

**Fig. 5:**
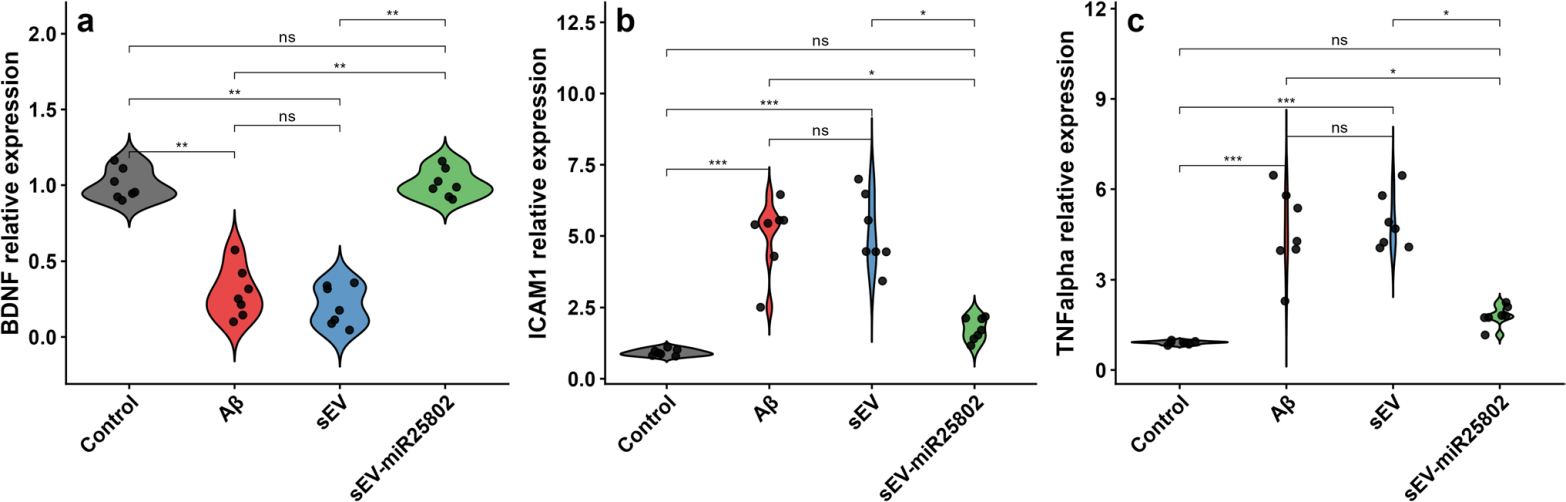
Effects of sEV‑miR25802 on Neuroinflammatory and Neuroprotective Gene Expression in Aβ-Induced Cells. Brain-derived neurotrophic factor (BDNF) mRNA levels (**a**), intercellular adhesion molecule 1 (ICAM1) mRNA levels (**b**), tumor necrosis factor alpha (TNF alpha/TNFα) mRNA levels (**c**). The mean ± SEM is used to express the data. Statistical comparisons were performed using the Kruskal–Wallis test (* *p* < 0.05, ** *p* < 0.01, *** *p* < 0.001, ns: not significant)

The levels of BDNF (Fig. 5a), ICAM1 (Fig. 5b), and TNF-α (Fig. 5c) (Supplementary Table 2) mRNA were compared between cells. According to the analysis results, ICAM1 and TNF-α gene expressions increased significantly in the Aβ-induced group compared to the control group, while BDNF mRNA levels decreased significantly. In contrast, a statistically significant decrease in the expression of TNF-α, and ICAM1 was observed in the sEV-miR25802-treated group compared to the Aβ-induced group, while a significant increase in BDNF expression was observed (*p* < 0.05). This research demonstrates that Aβ administration to cells triggers a neuroinflammatory response. Furthermore, sEV-miR25802 suppresses the Aβ-induced neuroinflammatory response and provide a neuroprotective effect through BDNF.

### Evaluation of BDNF Protein Levels by Western Blot Analysis

**Fig. 6:**
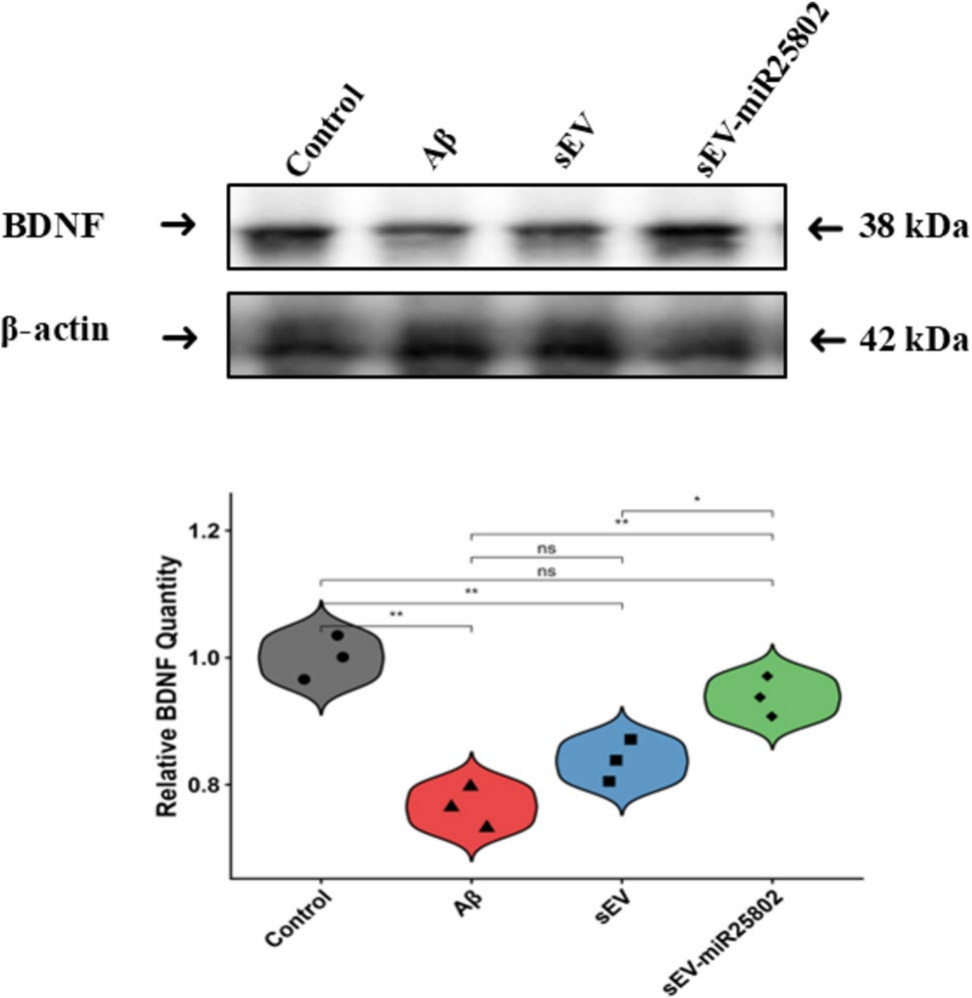
Effects of sEV-miR25802 on BDNF protein levels in Aβ-induced cells. Data are expressed as mean ± SEM. One-way ANOVA was used to assess statistical significance (* *p* < 0.05, ** *p* < 0.01, *** *p* < 0.001, ns: not significant)

BDNF protein levels were assessed by Western blot analysis. In the Aβ-stimulated group, a significant decrease in BDNF protein expression was observed; however, in the group treated with sEV-miR25802, BDNF levels rose to levels close to those of the control group (*p* < 0.05). The relative quantification values obtained after normalizing BDNF protein expression against β-actin are presented in Fig. 6. These findings demonstrate that sEV-miR25802 can restore the Aβ-induced decrease in BDNF levels to levels similar to those in the control group (*p* < 0.05).

### Evaluation of NfL, MIF, VEGF-A, and MCP-1 Protein Levels Using the ELISA Method

**Fig. 7:**
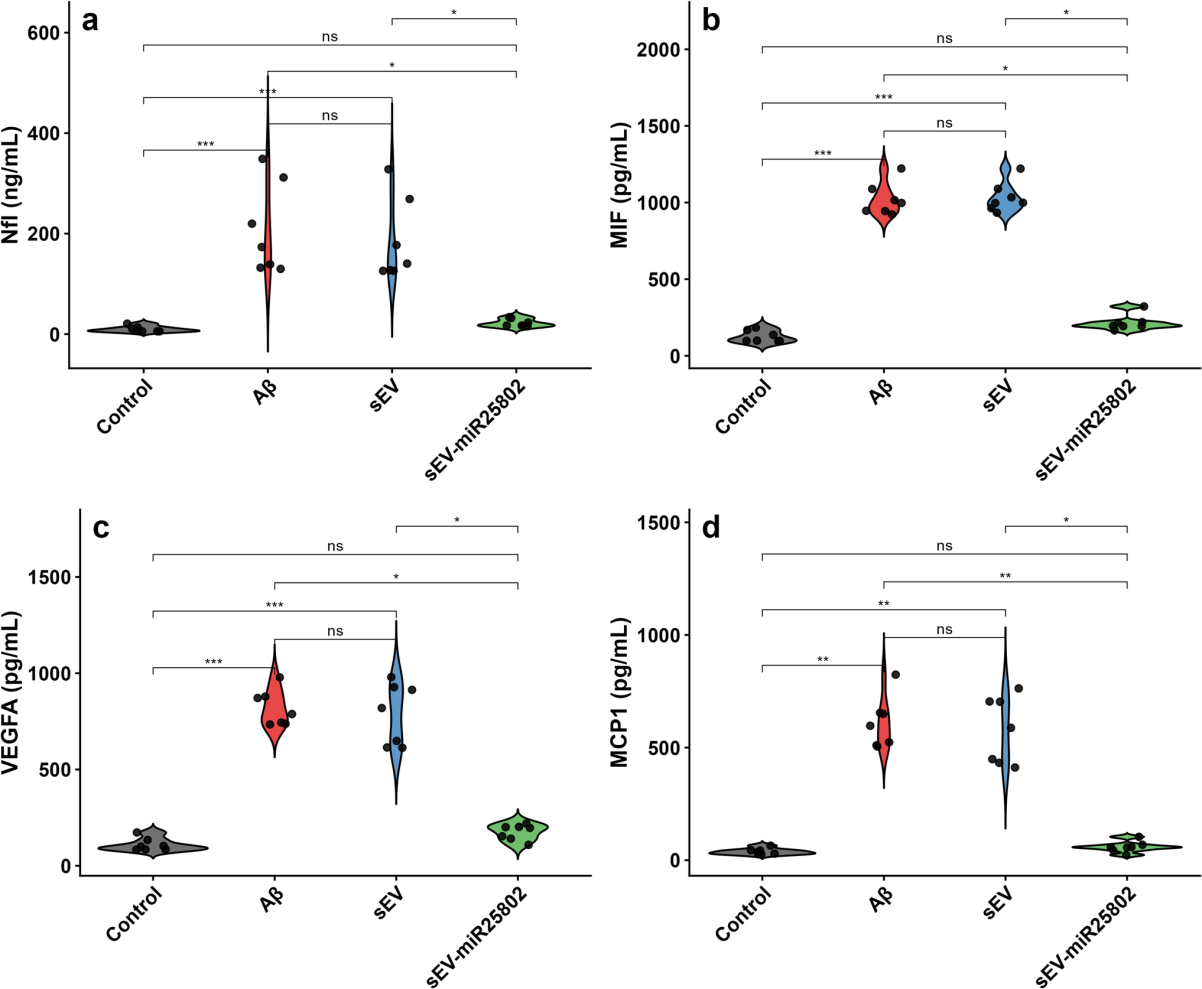
Effects of sEV‑miR25802 on Aβ-Induced Neuronal Damage and Neuroinflammatory Biomarkers. Protein levels of neurofilament light chain (NfL) (**a**), macrophage migration inhibitory factor (MIF) (**b**), vascular endothelial growth factor-A (VEGF-A) (**c**), and monocyte chemoattractant protein-1 (MCP-1) (**d**). The mean ± SEM is used to express the data. Statistical comparisons were performed using the Kruskal–Wallis test (* *p* < 0.05, ** *p* < 0.01, *** *p* < 0.001, ns: not significant)

The levels of NfL (Fig. 7a), MIF (Fig. 7b), VEGF-A (Fig. 7c), and MCP-1 (Fig. 7d) were evaluated using the ELISA method. As seen in Fig. 7, a significant increase was observed in the levels of NfL, MIF, VEGF-A, and MCP-1 in the Aβ-induced group compared to the control group (Supplementary Table 2). However, the sEV-miR25802-treated group showed a significant decrease in NfL, MIF, MCP-1, and VEGF-A levels compared to the Aβ-induced group (*p* < 0.05). These findings indicate that sEV-miR25802 attenuated Aβ-induced neuronal injury and neuroinflammation, while neuronal damage and neuroinflammation were increased in the Aβ-induced group. The results are consistent with previously reported effects of miR-25802 in promoting cell integrity and suppressing the neuroinflammatory response.

### Assessment of Cyt-c, TFAM, PINK1 and DNM1L Protein Levels Using the ELISA Method

**Fig. 8:**
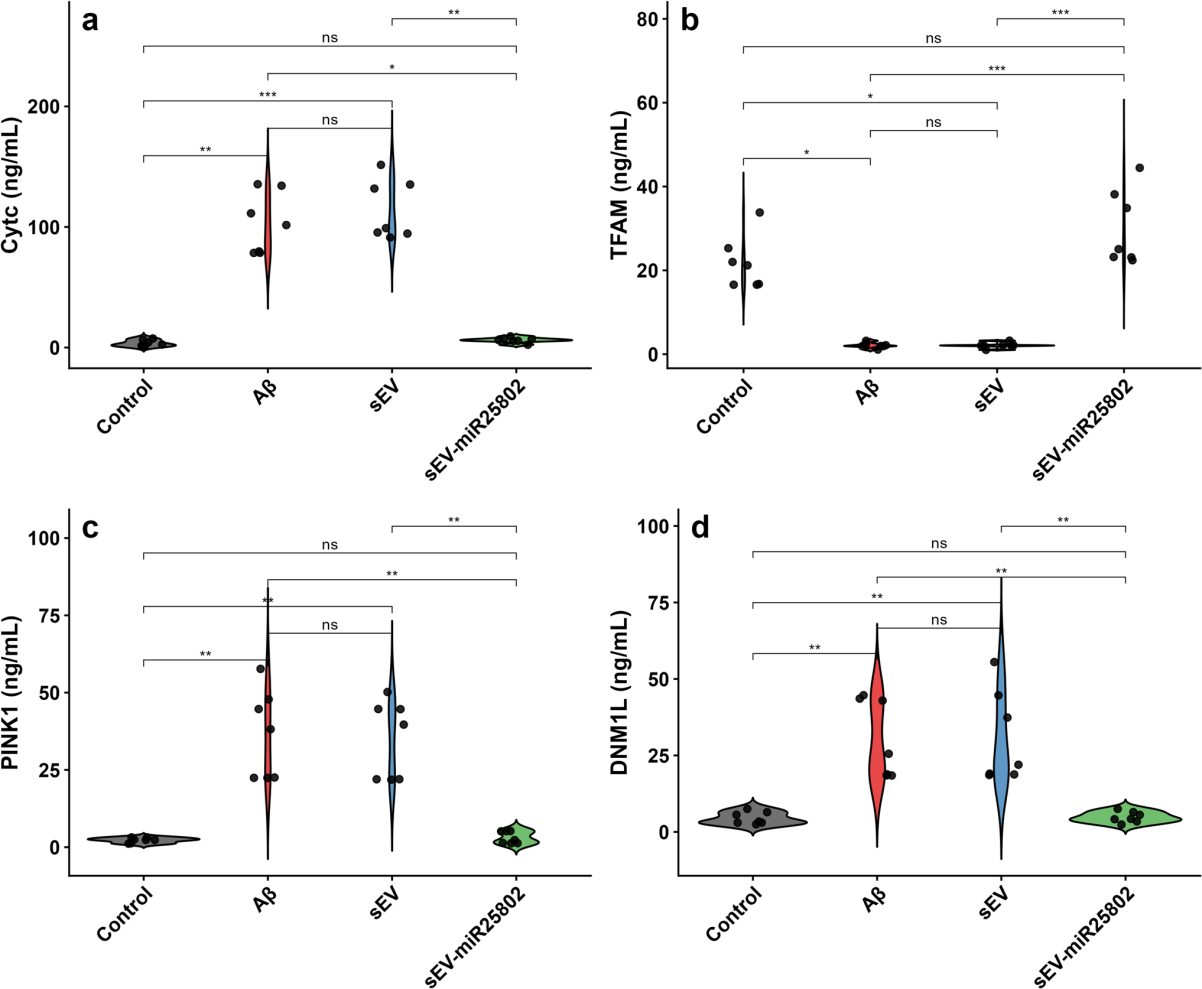
Effects of sEV‑miR25802 on Aβ-Induced Mitochondrial Dysfunction and Oxidative DNA Damage. Protein levels of cytochrome c (Cyt-c) (**a**), mitochondrial transcription factor A (TFAM) (**b**), PTEN-induced kinase 1 (PINK1) (**c**), dynamin-1-like protein (DNM1L) (**d**). The mean ± SEM is used to express the data. Statistical comparisons were performed using the Kruskal–Wallis test (* *p* < 0.05, ** *p* < 0.01, *** *p* < 0.001, ns: not significant)

The levels of Cyt-c (Fig. 8a), TFAM (Fig. 8b), PINK1 (Fig. 8c), and DNM1L (Fig. 8d) were evaluated using the ELISA method. In the Aβ group, Cyt-c, PINK1, and DNM1L levels were significantly increased compared to the control group, while TFAM levels were decreased (Supplementary Table 2). These results indicate that Aβ increases oxidative DNA damage by disrupting mitochondrial integrity. However, in the group treated with sEV-miR25802, a significant increase in TFAM levels and a significant decrease in Cyt-c, PINK1, and DNM1L levels were observed compared to the Aβ-induced group (*p* < 0.05). These results indicate that sEV-miR25802 improves mitochondrial damage by reducing oxidative DNA damage and Aβ-induced mitochondrial dysfunction.

### Evaluation of Expression Levels of CPLX2 and ROR1 Proteins

**Fig. 9:**
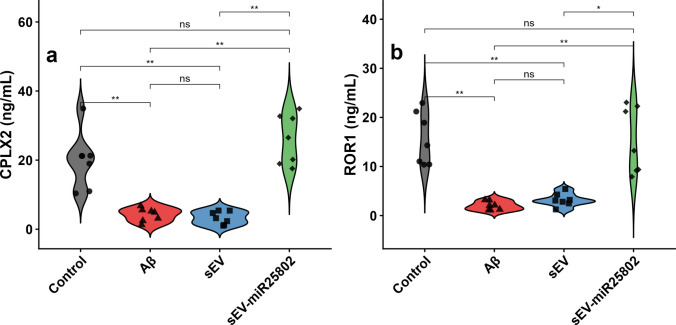
Effects of sEV-miR25802 treatment on synaptic and signaling-related protein levels in Aβ-induced neuroblastoma cells. Protein levels of complexin 2 (CPLX2) (**a**), and receptor tyrosine kinase-like orphan receptor 1 (ROR1) (**b**). The mean ± SEM is used to express the data. Statistical comparisons were performed using the Kruskal–Wallis test (* *p* < 0.05, ** *p* < 0.01, *** *p* < 0.001, ns: not significant)

As shown in Fig. 9, in the Aβ-induced group, CPLX2 and ROR1 levels were found to be significantly reduced compared to the control group (Supplementary Table 2) (*p* < 0.01). However, in the group treated with sEV-miR25802, a statistically significant increase in CPLX2 (Fig. 9a) and ROR1 (Fig. 9b) levels was observed compared to the Aβ-induced group (*p* < 0.01). These findings demonstrate that sEV-miR25802 protects neuronal integrity by controlling Aβ-induced synaptic protein loss.

### Evaluation of Aβ, Phosphorylated Tau (p-Tau181 and p-Tau217), and Total Tau Protein Levels

**Fig. 10:**
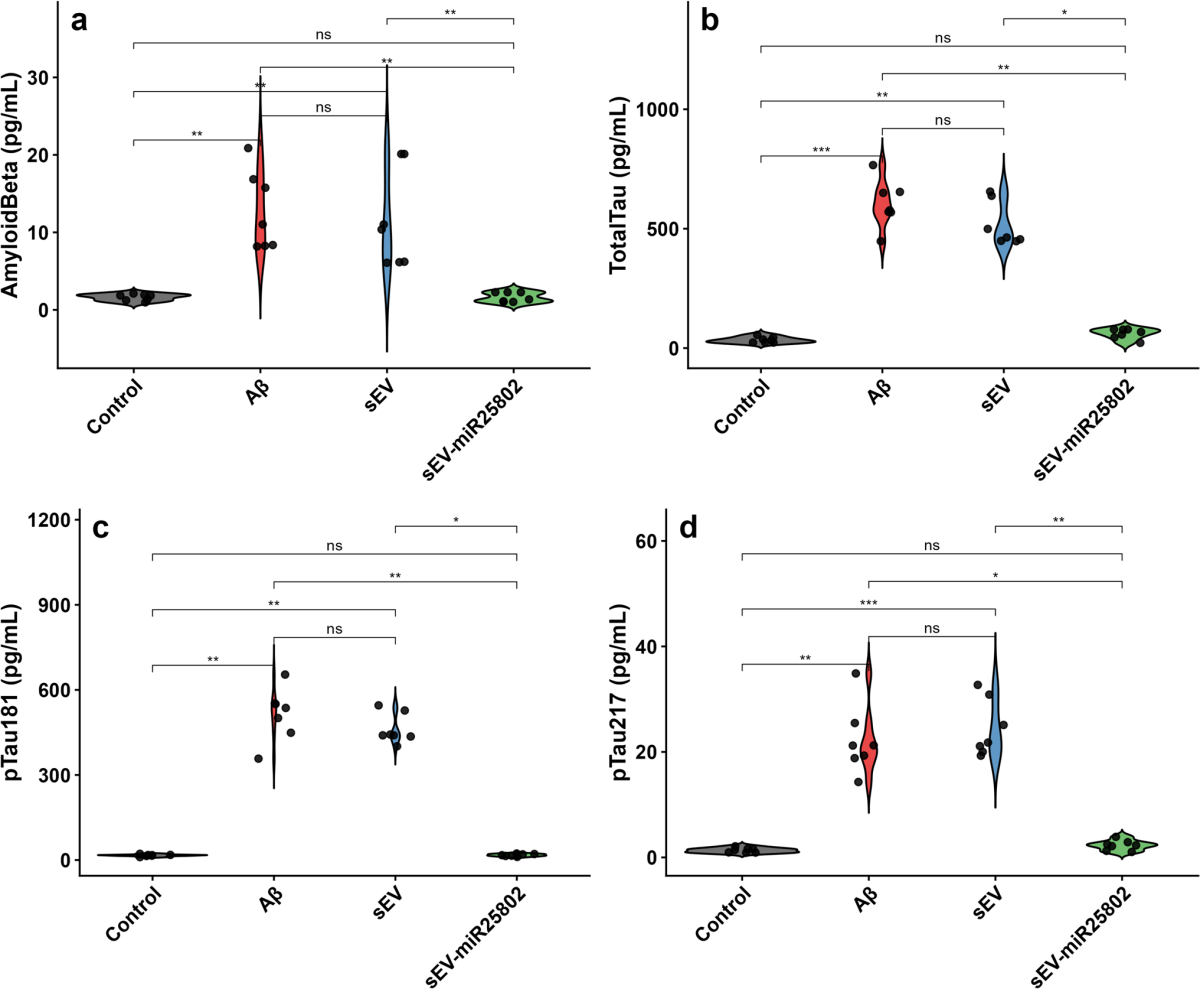
Effects of sEV-miR25802 on Tau Phosphorylation and Aβ Accumulation in Aβ-Induced Cells. Amyloid beta 1–40 (Aβ 1–40) (**a**), total Tau (**b**), phosphorylated Tau-181 (pTau-181) (**c**), phosphorylated Tau-217 (pTau-217) (**d**). The mean ± SEM is used to express the data. Statistical comparisons were performed using the Kruskal–Wallis test (* *p* < 0.05, ** *p* < 0.01, *** *p* < 0.001, ns: not significant)

According to the findings, Aβ (Fig. 10a), total Tau (Fig. 10b), p-Tau181 (Fig. 10c) and p-Tau217 (Fig. 10d) levels were found to be significantly increased in the Aβ-stimulated group compared to the control group (Supplementary Table 2). These results suggest that Aβ exposure triggers tau hyperphosphorylation, thereby activating neurodegenerative processes. However, a significant decrease in total Tau, p-Tau181, p-Tau217 and Aβ levels was seen in the sEV-miR25802-treated group compared to the Aβ-induced group (*p* < 0.05). This reduction suggests that sEV-miR25802 treatment provides a neuroprotective effect by suppressing Aβ-induced tau phosphorylation and amyloid deposition.

## Discussion

AD is characterized by progressive neuronal loss resulting from several pathogenic mechanisms, including mitochondrial dysfunction, oxidative stress, and neuroinflammation [38]. Recent studies have found that the upregulation of the Aβ protein is closely associated with oxidative stress damage [39, 40] and neuroinflammation [41]. However, while previous studies have addressed mechanisms such as mitochondrial dysfunction, oxidative stress, and neuroinflammation, it remains to be fully investigated whether miRNAs regulate AD pathogenesis holistically by simultaneously targeting these processes. It has been demonstrated that miRNAs may be crucial in the management of AD by concurrently controlling target genes that influence neurodegenerative processes. This study aimed to demonstrate that delivering miRNA-25802 to target cells via milk-derived sEVs in vitro reduces mitochondrial damage, oxidative stress, and inflammation. The findings indicate that miR-25802 has the ability to regulate important mechanisms involved in the development of AD. These results are consistent with the existing literature suggesting that miRNAs may have the potential to regulate AD pathogenesis [42, 43].

Following the characterization of milk-derived sEVs, this study examined the effects of miRNA-25802-loaded sEVs on mitochondrial damage, oxidative stress, and neuroinflammatory processes after delivery to target cells. Our findings suggest that miRNA-25802 plays a regulatory role in pathological processes associated with AD.

EVs are shown in the literature as potential therapeutic tools and biomarkers in the diagnosis and management of neurodegenerative diseases [44, 45]. This therapeutic potential is based on the ability of EVs to transport miRNAs, which play a role in intercellular communication, to recipient cells. Transported miRNAs play a critical role in regulating cellular signaling pathways and neurodegenerative processes [46, 47].

In approaches where exosomes are used as nanocarriers to transport therapeutic payloads, determining an effective loading method for miRNA and other nucleic acids is of great importance [48]. Exogenous substances can be physically and chemically loaded into exosomes, according to recent research. Various techniques, including electroporation, sonication, freeze–thaw cycles, and saponin-based membrane permeabilization, are used for this purpose [49, 50]. The current study sought to control cellular responses by exogenously loading particular miRNAs into milk-derived exosomes and delivering them to target cells.

Saponin, one of these methods, interacts with cholesterol in the membrane to create transient pores, thereby facilitating the entry of hydrophilic molecules and, in some cases, nucleic acids into exosomes [49]. Previous studies have also demonstrated that nucleic acids can be successfully loaded into exosomes [51, 52]. Dynamic light scattering analyses performed in this study showed that exosomes did not change in size after saponin application, and miR-25802 loading did not affect the physical properties of sEVs. Furthermore, the expression levels of miR-25802 were significantly increased compared with the sEV alone group, indicating that miR-25802 was successfully loaded into sEVs.

Accumulation of Aβ leads to activation of tau protein kinases, triggering phosphorylation of microtubule-associated tau proteins, thus amplifying each other's toxic effects [53]. Cell culture studies have shown that Aβ exposure increases Aβ production and pTau levels (especially pTau-181 and pTau-217) in SH-SY5Y cells [54, 55]. In this study, total tau, pTau-181, pTau-217, and Aβ1–40 levels increased in SH-SY5Y cells with Aβ treatment. This increase indicates that tau hyperphosphorylation and Aβ accumulation, one of the hallmarks of AD, were successfully recreated in the cellular model. All these markers were significantly decreased by administration of sEV-miR25802. Additionally, the decrease in Aβ1–40 levels demonstrates the regulatory effects of miR-25802 on amyloid metabolism. These findings are consistent with previously reported miRNA-based therapeutic strategies [56, 57] and suggest that miR-25802 may represent a potential therapeutic candidate for AD.

Oxidative stress occurs when ROS production exceeds the capacity of the antioxidant defense system. ROS, which include free radicals like superoxide and hydroxyl radicals, are extremely reactive molecules [58]. Because of its high oxygen consumption [59], high lipid content [60], and comparatively weak antioxidant defenses, the brain is vulnerable to oxidative injury [61]. The detection of elevated levels of oxidative stress markers in AD suggests that oxidative stress contributes to the onset of the disease [59, 62]. Previous research has shown that certain miRNAs, e.g., miR-214-3p, have an oxidative stress-reducing effect [60]. In the present investigation, we found that SOD and GPX1 activities decreased while ROS, MDA, and LDH levels rose in the Aβ-induced group relative to the control group. In the sEV-miR25802 group, the elevated ROS, LDH, and MDA levels were reduced, while SOD and GPX1 activities increased. These findings demonstrate the oxidative stress-alleviating effect of miR-25802.

In AD, microglia are activated in response to Aβ deposition, and this activation plays a role in disease progression through both neuroprotective and neurotoxic effects [56, 61]. Following activation, they differentiate into M1 (pro-inflammatory) and M2 (anti-inflammatory/neuroprotective) phenotypes [63]. While excessive activation of M1 microglia increases inflammation and neurodegeneration in AD, M2 microglia support Aβ clearance, reduce inflammation, and exert a protective effect against neuronal damage [64].

In the literature, it has been demonstrated that the neuroinflammatory response initiated by microglial activation in the pathogenesis of AD accelerates neuronal damage through increased proinflammatory cytokines and suppression of anti-inflammatory mechanisms [65, 66]. In this process, the increase in proinflammatory cytokines, particularly TNF-α, has been associated with the activation of the NF-κB signaling pathway [67, 68]. In our study, TNF-α expression was significantly elevated after Aβ administration compared to the control group. In contrast, sEV-miR25802 treatment significantly suppressed the Aβ-induced increase in TNF-α. These findings suggest that miRNA-based strategies may be a therapeutic strategy to reduce neuroinflammation and delay the Aβ-induced neurodegenerative process.

BDNF is a key regulator of neuronal survival, synaptic plasticity, and cognitive function. It has been observed that BDNF levels decrease in AD, contributing to synaptic loss and neuronal degeneration [69, 70]. According to some studies, a decrease in BDNF levels accelerates tau hyperphosphorylation, Aβ accumulation, neuroinflammation, and neuronal death [71]. Therefore, we examined how sEV-miR25802 administration affected BDNF levels in Aβ-induced neuroblastoma cells. Our findings showed that BDNF expression was significantly decreased in Aβ-treated cells compared to the control group, while sEV-miR25802 treatment significantly increased the decreased BDNF levels. These results imply that by raising BDNF levels, miR-25802 may improve cell proliferation.

Cells in blood vessel walls and many other cell types produce the protein known as ICAM-1. It localizes to these cells' surfaces, allowing them to interact with nearby cells [72]. Under normal physiological conditions, ICAM-1 is expressed at low levels, but increases with the onset of inflammatory processes [73, 74]. In AD, peripheral immune cell infiltration and neuroinflammatory processes lead to increased ICAM-1 expression in endothelial cells [74]. In our study, ICAM-1 levels were significantly increased in the Aβ-stimulated group. Treatment with miR-25802, however, halted this rise and brought protein levels back to those of the control group. These results suggest that Aβ-induced neuroinflammation may be reduced by miR-25802.

The expression levels of additional inflammatory and vascular markers (NfL, MIF, VEGF-A, and MCP-1) that are involved in the pathophysiology of AD were also investigated in this work. Our findings showed that these parameters were significantly increased in Aβ-induced cells, but this increase was suppressed by sEV-miR25802 treatment. This finding suggests that this miRNA exerts a neuroprotective effect by controlling endothelial activation and inflammatory responses.

Synaptic loss and synaptic dysfunction are important pathological events occurring in the early stages of AD [75]. Synaptic dysfunction is closely associated with changes in presynaptic proteins [76]. Previous studies have reported that decreased CPLX2 levels in AD lead to synaptic transmission disorders and decreased cognitive functions [77, 78]. In this study, the levels of CPLX2, a presynaptic protein that regulates synaptic vesicle fusion and neurotransmitter release, were examined. In our study, the decrease in CPLX2 levels in cells treated with Aβ demonstrates that Aβ disrupts synaptic integrity and leads to synaptic dysfunction. In this study, use of sEV-miR25802 significantly increased the levels of CPLX2, which was reduced by Aβ. This increase indicated that miR-25802 prevented the cognitive impairment associated with increased expression of CPLX2, which plays a role in the regulation of synaptic dysfunction.

Cell surface membrane proteins known as receptor tyrosine kinases (RTKs) have the ability to autophosphorylate in response to external ligand cues and then send this signal downstream. Critical elements of neuronal development and physiology, including axonal growth cone guidance, synaptic signaling, and cell-to-cell communication, have been demonstrated to be regulated by RTKs [79]. ROR1 contributes to the upkeep of developmental processes and structural stability as a member of the ROR family. It has a special role in the development of the neurological system [80]. The current literature reports that ROR1 is generally decreased in Aβ models [81, 82]; consistent with these findings, we observed a decrease in ROR1 levels in our study. On the other hand, the increase in ROR1 levels in the group treated with miR-25802 suggests that it reduces oxidative stress and Aβ-dependent cellular damage in the cellular environment. In conclusion, the findings show that miR-25802 is effective in restoring cellular homeostasis through the ROR1-mediated survival mechanism.

Finally, an important pathological mechanism contributing to the progression of AD is mitochondrial dysfunction [2]. Mitochondria are essential for many biochemical processes, including respiratory functions and bioenergy, and are vital for supporting cell viability [83]. Fission and fusion mechanisms, which preserve mitochondrial quality and homeostasis, cause fast changes in mitochondrial morphology in response to external stimuli and shifts in metabolic state [84]. In the last few years, significant studies have been conducted investigating the impact of various mitochondrial dysfunctions, such as ROS production, mitochondrial Ca^2+^ imbalance, ATP loss, mitochondrial dynamics, transport defects and mitophagy, on the pathogenesis of AD [85, 86]. In the present study, increased levels of Cyt-c release, DNM1L, and PINK1 following Aβ administration indicate oxidative DNA damage and mitochondrial injury. Additionally, decreased TFAM levels suggest decreased mitochondrial transcriptional activity. sEV-miR25802 treatment significantly reversed these impairments and reduced mitochondrial damage. These findings suggest that miR-25802 alleviates Aβ-induced mitochondrial dysfunction.

## Limitations

Although this study provides comprehensive insight into the neuroprotective role of miR-25802 against Aβ-induced neuronal damage through the modulation of pathways related to oxidative stress, mitochondrial dysfunction, and neuroinflammation, several limitations should be acknowledged. First, all experiments were conducted using an in vitro Aβ-induced SH-SY5Y neuroblastoma cell model, which, while widely accepted for mechanistic investigations, does not fully recapitulate the cellular complexity, neuronal–glial interactions, and systemic features of AD observed in vivo. Consequently, the translational relevance of the findings may be limited and in vivo studies are needed to confirm the preventive effects of miR-25802 against synaptic dysfunction and cognitive impairment.

Second, although numerous markers associated with oxidative stress, inflammation, and mitochondria were quantified, protein expression was primarily examined using ELISA-based methods. Although BDNF expression has been validated by Western blot analysis, the absence of complementary protein validation techniques—such as Western blot or immunocytochemical analyses—for other proteins may limit the scope of protein-level characterization, particularly regarding post-translational modifications and subcellular localization.

Finally, this study focused on short-term cellular responses to sEV-miR25802 treatment. Long-term effects, dose-dependent dynamics, and potential off-target effects were not investigated. Additionally, while changes in CPLX2 levels suggest a potential role for miR-25802 in synaptic regulation, using a single synaptic marker is insufficient to represent synaptic function. Therefore, conclusions regarding the prevention of synaptic dysfunction and cognitive impairment should be interpreted with caution. Furthermore, in vivo validation and behavioral assessments were not investigated in this study. Future studies are needed that utilize additional synaptic and protein validation approaches, along with primary neuronal cultures, co-culture systems including glial cells, and in vivo AD models.

## Conclusions

In conclusion, we demonstrated in our study that miR-25802, a novel non-coding RNA sequence, was successfully loaded into small vesicles. It was shown that miR-25802 protects neurons from Aβ-induced damage by modifying genes related to oxidative stress, mitochondrial damage, and neuroinflammation mediated by microglia. These results suggest that miR-25802 is a targetable molecule in the treatment of AD.

## Supplementary Information

Below is the link to the electronic supplementary material.

Supplementary file1 (DOCX 239 KB)

Supplementary file2 (XLSX 13 KB)

Supplementary file3 (XLSX 24 KB)

## Data Availability

Data will be made available on request.
