# Supplementary figures and images for: Delivery of miR-25802 via Small Vesicles Protects Against Mitochondrial Injury, Oxidative Stress, and Neuroinflammation in Alzheimer’s Disease
Source: Mol Neurobiol. 2026 Apr 23;63(1):583. doi: 10.1007/s12035-026-05889-7 (PMC13102934; doi:10.1007/s12035-026-05889-7)

**Melting Curve graphics are provided at Supplementary Figure 1.**


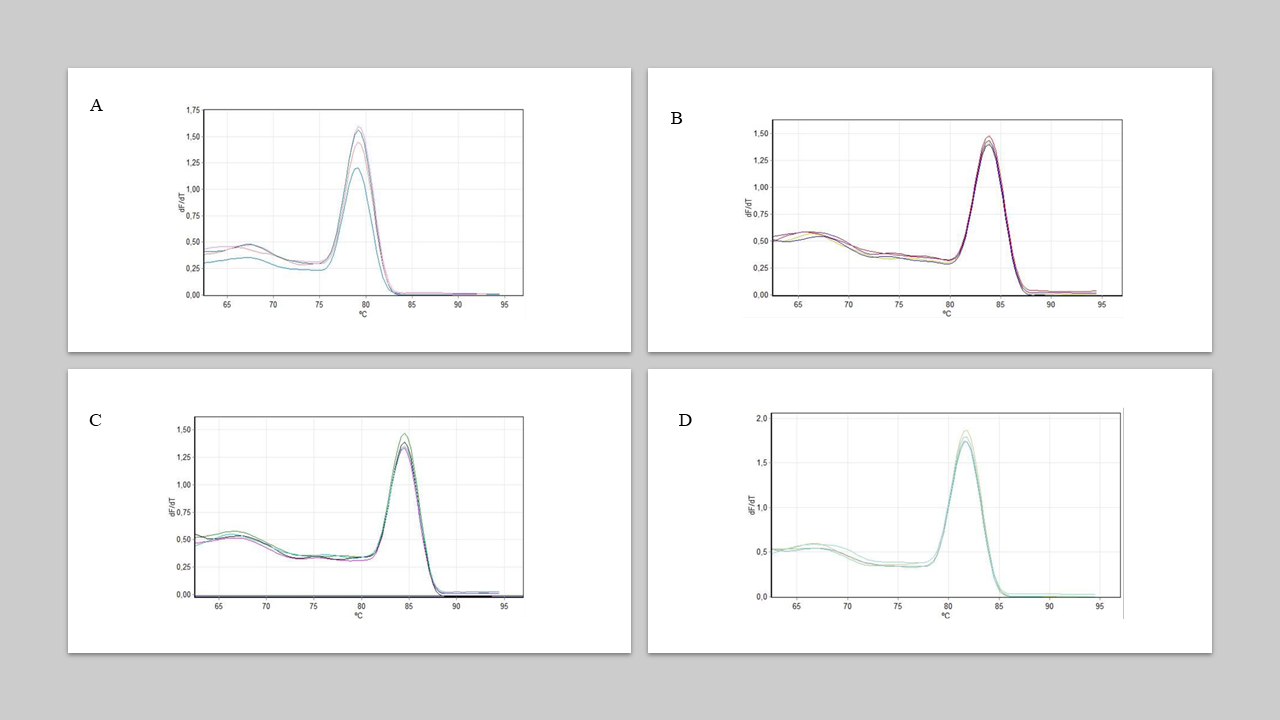


**A. BDNF, B.TNF-α, C. ICAM1, D. GAPDH**

Supplement: Supplementary file 1 — Supplementary file1 (DOCX 239 KB) [file 12035_2026_5889_MOESM1_ESM.docx]
